# Performance Analysis of GNSS/INS Loosely Coupled Integration Systems under Spoofing Attacks

**DOI:** 10.3390/s18124108

**Published:** 2018-11-23

**Authors:** Rui Xu, Mengyu Ding, Ya Qi, Shuai Yue, Jianye Liu

**Affiliations:** Navigation Research Center, Nanjing University of Aeronautics and Astronautics, Nanjing 210016, China; mengyuding@nuaa.edu.cn (M.D.); nuaaqiya@nuaa.edu.cn (Y.Q.); yueshuai123@nuaa.edu.cn (S.Y.); ljyac@nuaa.edu.cn (J.L.)

**Keywords:** IMU error compensations, Kalman filter, integration system, GNSS, INS, GNSS spoofing interference

## Abstract

The loosely coupled integration of Global Navigation Satellite System (GNSS) and Inertial Navigation System (INS) have been widely used to improve the accuracy, robustness and continuity of navigation services. However, the integration systems possibly affected by spoofing attacks, since integration algorithms without spoofing detection would feed autonomous INSs with incorrect compensations from the spoofed GNSSs. This paper theoretically analyzes and tests the performances of GNSS/INS loosely coupled integration systems with the classical position fusion and position/velocity fusion under typical meaconing (MEAC) and lift-of-aligned (LOA) spoofing attacks. Results show that the compensations of Inertial Measurement Unit (IMU) errors significantly increase under spoofing attacks. The compensations refer to the physical features of IMUs and their unreasonable increments likely result from the spoofing-induced inconsistency of INS and GNSS measurements. Specially, under MEAC attacks, the IMU error compensations in both the position-fusion-based system and position/velocity-fusion-based system increase obviously. Under LOA attacks, the unreasonable compensation increments are found from the position/velocity-fusion-based integration system. Then a detection method based on IMU error compensations is tested and the results show that, for the position/velocity-fusion-based integration system, it can detect both MEAC and LOA attacks with high probability using the IMU error compensations.

## 1. Introduction

The vulnerability of Global Satellite Navigation Systems (GNSSs) to various intentional and non-intentional radio frequency interferences is an obstacle of GNSS applications [1,2]. Within them, the spoofing interference is a type of troublesome and malicious interference. Spoofing signals have the same characteristics to the legitimate GNSS signals. Thus, they are able to pass through the correlators of target receivers. Usually stronger than authentic signals, spoofing signals guide the victim receivers to track themselves, and then throw the victims astray [3,4,5].

Many approaches have been proposed to detect or suppress spoofing attacks. For stand-alone GNSS receivers, multiple antennas are widely used to mitigate the effect of spoofing interference by monitoring the direction of arrival signals [6,7]. For single-antenna GNSS receiver, some signal-processing-based techniques have been implemented as effective ways to find spoofing attacks, including Receive Power Monitoring (RPM) [8], correlation function analysis [9] and Kalman filter-based tracking loop [10].

Besides, INS aided methods have also developed because the inertial navigation system (INS) is autonomous and the integration of GNSS and INS is considered the possibility of countering spoofing attacks. The GNSS/INS integration systems overcome the drawbacks of each stand-alone system and become popular navigation systems [11,12,13]. In the integration systems, the defects of INSs, the unknown absolute initial position and the accumulative position errors are compensated by GNSS that provides absolute positioning estimations with stationary noise [11,14,15]. Meanwhile, autonomous INSs independent of surroundings have the capability to improve the robustness of GNSSs [16,17].

Actually, of normal GNSS/INS integration systems, the capability to counter spoofing attacks is limited [18,19,20], since spoofing effects on GNSS probably pollute the estimations of the integration algorithm, such as the Kalman Filter, and then mis-correct the INS states. Some current researches improve the performance of integration systems under spoofing attacks. Using an INS-aided integrity monitoring algorithm, the tightly GNSS/INS integration systems, which fuse both systems using pseudoranges, effectively mitigate spoofing attacks [21]. INS-estimated positions, as well as the satellite positions and receiver clock errors from the GNSS, are used to generate redundant virtual pseudoranges, considered as spoofing-free reference pseudoranges. When one satellite pseudorange is quite different from its virtual pseudorange, the distribution of pseudorange residuals change and the satellite is likely under spoofing attacks. Besides, as the Kalman Filter innovations in tightly GNSS/INS integration systems show unreasonable fluctuations under spoofing environments, they can be used as an alternative detection method [22,23,24]. With dual antennas, GNSS is able to measure the vehicle heading. The consistency of attitudes resolved by INS and GNSS is able to detect spoofing attacks, due to the difference from the spoofing heading to the actual heading [25,26].

However, these methods are difficultly realized in low-cost GNSS chips that support neither pseudorange outputs nor dual/multiple antenna inputs. Additionally, for these black-box receivers, there is no access to signal processing-based interference detections. The GNSS/INS loosely coupled integration system, fusing both systems using positions (and velocities in some cases), is easily available and widely employed [20]. Thus, we try to find a method to detect spoofing attacks based on the GNSS/INS loosely coupled integration system without additional hardware, information or special requirements.

Considering different behaviors of spoofing effects and different integration fusions, we analyze the performance of the GNSS/INS loosely coupled integration systems with position and position/velocity fusion under two typical spoofing attacks, Meaconing attacks (MEAC attacks) with constant spoofing-induced relative pseudoranges and Lift-off-aligned attacks (LOA attacks) with gradually increasing relative pseudoranges [27]. In the analysis, we focus on variations of the IMU error compensations estimated by the Kalman filer. The compensations refer to IMU physical characteristics and vary within reasonable ranges. Abnormal increments of compensations are likely to alarm spoofing attacks and deeply discussed in the study. Besides, a spoofing detection algorithm based on the compensations is proposed and tested. MEAC attack could be alarmed by GNSS/INSs with both position and position/velocity fusion within one second and two seconds, respectively. For LOA attack, GNSS/INSs with position/velocity fusion are capable of alarming it instantly, while the system with position fusion fails in perceiving spoofing interference. One obvious merit of the spoofing detection is the easy availability for low-cost GNSS/INSs without extra-hardware or improvement on the receiver structure.

In Section 2, the main features of different types spoofing attacks are introduced, as well as their effects on GNSS position and velocity estimations. In Section 3, the effects of spoofing interferences on GNSS/INS loosely coupled integration system are discussed in detail. The effects of different spoofing interferences are compared by experimental studies and a detection method of spoofing attacks is proposed in Section 4. Finally, the conclusions of this study are given in Section 5.

## 2. Effects of Spoofing Attacks on GNSS Position and Velocity Estimations

Spoofing attacks can be realized by using special devices, such as a GNSS transmitter, to emit GNSS-like signals. The GNSS-like signals have the same signal structures to the authentic signals, but high signal power and different PRN code delays $\tau_{s}$. The similar signal structures and high power cause the spoofing signals passing through the correlators and being tracked by the receiver. The different PRN code delays induce different pseudorange measurements and then the false GNSS position and velocity estimations. The delay $\tau_{s}^{i}$ of spoofing signal referring to the i-th satellite can be written as:

(1) $$\tau_{s}^{i}=\tau_{a}^{i}+∆\tau^{i},$$

where $\tau_{a}^{i}$ is the delay of the authentic signal from the i-th satellite and $∆\tau^{i}$ is the relative spoofing delay. Correspondingly, the relationship within spoofing, authentic and relative pseudoranges is written as:

(2) $$\rho_{s}=c\tau_{s}=\rho_{au}+∆\rho ={\left[\rho_{au}^{i}\right]}_{N\times 1}+{\left[∆\rho_{s}^{i}\right]}_{N\times 1},$$

where c is the speed of light, $\rho_{s}$ and $\rho_{au}$ are spoofing and authentic pseudorange vectors, respectively, and *N* is the number of available satellites. The estimated position $P_{GNSS}$ and receiver clock bias $\delta t_{u}$ are

(3) $$\left[\begin{array}{c} P_{GNSS}\\ \delta t_{u} \end{array}\right]={\left(A^{T}A\right)}^{-1}A^{T}\left[\rho_{au}+∆\rho \right]={\left(A^{T}A\right)}^{-1}A^{T}\rho_{au}+{\left(A^{T}A\right)}^{-1}A^{T}∆\rho =\left[\begin{array}{c} P_{au}+∆p_{s}\\ \delta t_{ua}+∆\delta t_{us} \end{array}\right],$$

where A is the satellite geometry matrix referring to the satellite number and distribution, $P_{au}$ is the authentic position, $∆p_{s}$ is the spoofing induced relative position, $\delta t_{ua}$ is the authentic receiver clock bias, and $∆\delta t_{us}$ is the spoofing induced relative receiver clock bias.

Meanwhile, the simulated motion of the spoofing signals also leads to incorrect interpretation of the Doppler shift ($f_{s}^{i}\neq f_{d}^{i}\;\left[\mathrm{Hz}\right]$). The false Doppler shifts mis-lead the velocity solution of the victim receiver. The relationship between the Doppler shift and pseudorange rate is expressed as:

(4) $${\dot{\rho }}_{s}^{i}={\dot{\rho }}_{au}^{i}+∆{\dot{\rho }}_{s}^{i}=-\lambda \left(f_{d}^{i}+∆f_{s}^{i}\right)=-\lambda f_{s}^{i},$$

Similarly, the velocity solution can be written as

(5) $$\left[\begin{array}{c} V_{GNSS}\\ \delta {\dot{t}}_{u} \end{array}\right]={\left(A^{T}A\right)}^{-1}A^{T}\left(-\lambda f_{s}\right)=-{\left(A^{T}A\right)}^{-1}A^{T}\left(\lambda f_{d}\right)+{\left(A^{T}A\right)}^{-1}A^{T}∆{\dot{\rho }}_{s}=\left[\begin{array}{c} V_{au}+∆v_{s}\\ \delta {\dot{t}}_{ua}+\delta {\dot{t}}_{us} \end{array}\right],$$

where $V_{au}$ is the velocity vector from the authentic signals, $∆v_{s}$ is the spoofing induced relative velocity, $\delta {\dot{t}}_{ua}$ is the authentic receiver clock error rate, and $\delta {\dot{t}}_{us}$ is the spoofing induced relative receiver clock drift.

**Figure 1:**
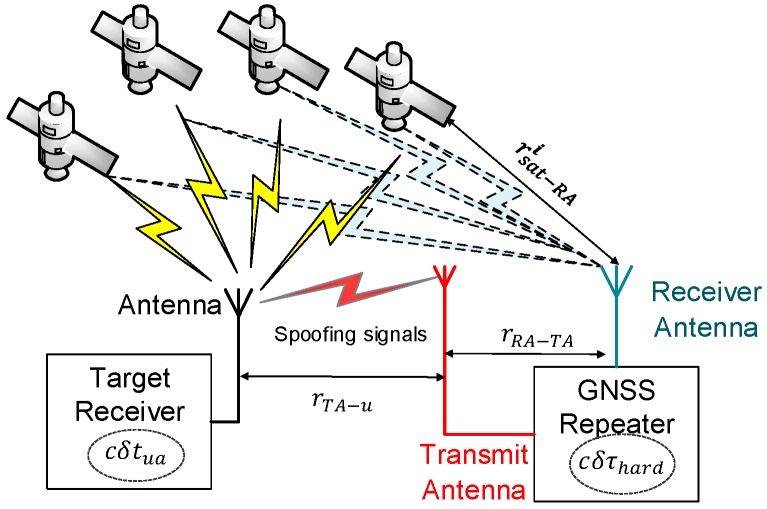
A schema of transmitter-based spoofing attack.

Spoofing attacks can be simply and low-costly realized by a transmitter or a repeater which delay the received GNSS signals and transmit them with high power via a transmitting antenna, as shown in Figure 1. Under the situation, signals collected by the target receiver are transmitted from the spoofer rather than the satellites. Therefore, the pseudoranges measured by the target receiver consist of the ranges from satellites to the spoofer receiving antenna $r_{sat-RA}^{i}$, from the spoofer receiving antenna to the spoofer transmitting antenna $r_{RA-TA}$, and from the spoofer transmitting antenna to the target receiver $r_{TA-u}$. In addition, the measured pseudoranges include the hardware-time-delay induced range $c\delta \tau_{hard}$ in the spoofer and receiver-clock-bias induced range $c\delta t_{ua}$ in the target receiver.

Within these ranges, $r_{sat-RA}^{i}$ is different in each pseudorange $\rho_{s}^{i}$ since its value refers to the different satellite position. The ranges $r_{RA-TA}$, $r_{TA-u}$, $c\delta \tau_{hard}$ and $c\delta t_{ua}$ are the same in all pseudoranges. The total pseudorange collected by the target receiver referring to one satellite is written as,

(6) $$\rho_{s}^{i}=r_{sat-RA}^{i}+r_{RA-TA}+r_{TA-u}+c\delta \tau_{hard}+c\delta t_{ua}.$$

From Equations (2) and (6), the relative spoofing pseudorange can be estimated as,

(7) $$\Delta \rho^{i}=r_{sat-RA}^{i}-r_{au}^{i}+r_{RA-TA}+r_{TA-u}+c\delta \tau_{hard}.$$

Equation (7) shows that the spoofing induced position error $∆p_{s}$ depends on the range difference between $r_{sat-RA}^{i}$ and $r_{au}^{i}$. The spoofing induced clock bias error $\delta t_{us}$ (in the unit of meter) includes $r_{RA-TA}$, $r_{TA-u}$, $c\delta \tau_{hard}$ and the common part of $r_{sat-RA}^{i}-r_{au}^{i}$. Each them has the same value in all pseudoranges and hence the same behavior to the receiver clock bias. Specially, if $r_{sat-RA}^{i}=r_{au}^{i}$ and $r_{TA-u}=0$, namely the transmitter and the target receiver settled near to each other, it can be obtained that $∆p_{s}=0$.

In Equation (7), the item $r_{RA-TA}$ is constant, equal to the cable length between the spoofer receiving antenna and transmitting antenna. Other items $r_{sat-RA}^{i}$, $r_{au}^{i}$, $r_{TA-u}$ and $\delta \tau_{hard}$ are time-varying. Thus, the spoofing induced pseudorange rate is written as,

(8) $$\Delta {\dot{\rho }}^{i}={\dot{r}}_{sat-RA}^{i}-{\dot{r}}_{au}^{i}+{\dot{r}}_{TA-u}+c\delta {\dot{\tau }}_{hard}.$$

Similarly, the different part ${\dot{r}}_{sat-RA}^{i}-{\dot{r}}_{au}^{i}$ in the pseudorange rate leads to velocity variation, which is the spoofing induced velocity. The common parts ${\dot{r}}_{TA-u}$ and $c\delta {\dot{\tau }}_{hard}$ affect the receiver clock drift error.

Neither receiver clock bias nor drift is integrated in the GNSS/INS loosely coupled integration system. Their variations under spoofing attacks are simplified in the following analysis. According to Equations (3), (5), (7) and (8), it can be obtained that $\Delta p_{s}=\Delta v_{s}t+\Delta p_{s0}$. A common case of $\Delta v_{s}=0$ and $\Delta p_{s0}\neq 0$ would occur under MEAC attack. The item $\Delta p_{s0}$ is the initial position offset, the position difference between the spoofer and the target receiver. The nonzero item $\Delta v_{s}$ results from the pseudorange rate difference of ${\dot{r}}_{sat-RA}^{i}$ and ${\dot{r}}_{au}^{i}$ When the spoofer is near to the target receiver and both are relatively static, the item $\Delta v_{s}$ is close to zero. The value of $\Delta p_{s}$ steps up to a constant value when spoofing occurs.

Different to the impulsive position error case, the other common case of $\Delta v_{s}\neq 0$ and $\Delta p_{s0}=0$ would occur under Lift-off-aligned attack (LOA attack). In this case, the spoofing signal aligned to the authentic signal at the beginning of the attack, i.e., $\Delta \rho^{i}=0$, meanwhile the spoofing induced position deviation $\Delta p_{s0}=0$. Under LOA attack, the spoofing relative delay increases with time. As a result, the spoofing induced relative pseudorange $\Delta \rho^{i}$ increases with time, i.e., $\Delta \rho^{i}=\Delta {\dot{\rho }}^{i}t$. In this case, pseudorange rate is nonzero and the position estimated from the spoofed receiver are gradually away from the authentic position over time, i.e., $\Delta p_{s}=\Delta v_{s}t$. It should be noted that the variation $\Delta p_{s}=\Delta v_{s}t$ is also able to realize under MEAC attack. For instance, the spoofer is close to the target receiver and then far away from it. The simple implementation is not exact LOA attack since the spoofing signal with unknown hardware delay is unaligned to the authentic signal.

Generally, the position and velocity measured by GNSS under spoofing attacks can be written as,

(9) $$\begin{array}{c} \left[\begin{array}{c} P_{GNSS}\\ V_{GNSS} \end{array}\right]=\left[\begin{array}{c} P_{au}+\Delta p_{s}\\ V_{au}+\Delta v_{s} \end{array}\right],\\ \Delta p_{s}=\Delta v_{s}t+\Delta p_{s0}. \end{array}$$

Shortly, under MEAC attack, $\Delta v_{s}=0$ and $\Delta p_{s0}\neq 0$, GNSS-estimated positions step up without significant velocity variations. Under LOA attack, $\Delta v_{s}\neq 0$ and $\Delta p_{s}=\Delta v_{s}t$, GNSS-estimated velocities jump up and the estimated positions are gradually away from the actual position.

## 3. Effects of Spoofing Attacks on the GNSS/INS

The variations of spoofing-induced relative positions and velocities under MEAC and LOA attacks can be described as two typical situations of $\Delta p_{s}=\Delta p_{s0}$ and $\Delta p_{s}=\Delta v_{s}t$, respectively. Their effects on the integration system are investigated after a short introduction to the integration system model under spoofing-free situation.

### 3.1. The GNSS/INS Loosely Coupled Integration System Model in the Normal Case

GNSS/INS loosely coupled integration systems commonly employ the Kalman Filter to estimate the position and velocity errors, gyroscope bias and first-order Markov process random noise errors, and accelerometer bias error. Then, the state vector $X_{k}={\left[\begin{array}{ccc} \delta P_{k} & \delta V_{k} & \begin{array}{ccc} \varepsilon_{b,k} & \varepsilon_{r,k} & \nabla_{k} \end{array} \end{array}\right]}^{T}$ consists of INS position error δP, velocity error δV, gyroscope bias errors $\varepsilon_{b}$, and first-order Markov process random noise errors of gyroscope $\varepsilon_{r}$ and accelerometer bias error ∇ [2,6,28]. The process model is commonly as,

(10) $$X_{k}=F_{k,k-1}X_{k-1}+G_{k-1}W_{k-1},$$

where $F_{k,k-1}$ is the state transition matrix, $G_{k}$ is the system noise matrix, and $W_{k}$ is the process noise, which is assumed as white noise with covariance $Q_{k}=E\left[W_{k}W_{k}^{T}\right]$.

The acquisition of attitude in GNSS requires multiple antennas, while typical commercial GNSS receivers are equipped with only one receiver antenna and cannot resolve attitudes directly. Therefore, in the GNSS/INS loosely coupled integration systems, GNSS position or GNSS position/velocity are fused with INS. The position and velocity differences between INS and GNSS are considered as measurements. The measurement model is written as:

(11) $$Z_{k}=\left[\begin{array}{c} Z_{p,k}\\ Z_{v,k} \end{array}\right]=\left[\begin{array}{c} P_{INS,k}-P_{GNSS,k}\\ V_{INS,k}-V_{GNSS,k} \end{array}\right]=\left[\begin{array}{c} \delta P_{k}+N_{g,k}\\ \delta V_{k}+M_{g,k} \end{array}\right]=\left[\begin{array}{c} H_{p,k}\\ H_{v,k} \end{array}\right]X_{k}+\left[\begin{array}{c} N_{g,k}\\ M_{g,k} \end{array}\right]=H_{k}X_{k}+V_{k},$$

where $N_{g}$ and $M_{g}$ are position error and velocity error of GNSS, respectively. $H_{p}$, $H_{v}$ are the measurement matrices. The measurement error $V_{k}$ is considered as white noise, i.e., $E\left(V_{k}\right)=0$. Its covariance $R_{k}$ can be estimated as $R_{k}=E\left[V_{k}V_{k}^{T}\right]$. Details about the specific parameters of F,G,H can be found in [28]. In Equation (11), the item $Z_{k}$ represents the position and velocity difference between the GNSS measurements and INS estimation. Under the normal cases, the position and velocity states of GNSS are consistent with that of INS, and hence the position and velocity difference $Z_{k}$ is small, equal to the sum of GNSS noise and INS errors, i.e., $Z_{k}=V_{k}$. If all INS errors are corrected, the item $Z_{k}$ is GNSS noise. In some loosely coupled integration systems, the velocity difference δV is optional. Then, the GNSS/INS integration system becomes integration with position fusion, and the measurement model becomes $Z_{k}=H_{p,k}X_{k}+N_{g,k}$.

### 3.2. Analysis of the Effect of Spoofing Interference on the GNSS/INS

Kalman Filter is most used in the GNSS/INS integration systems. In a standard Kalman Filter, the posteriori state estimate ${\hat{X}}_{k}$ is estimated as,

(12.1) $${\hat{X}}_{k}={\hat{X}}_{k,k-1}+K_{k}\left(Z_{k}-H_{k}{\hat{X}}_{k,k-1}\right),$$

(12.2) $${\hat{X}}_{k,k-1}=F_{k,k-1}{\hat{X}}_{k-1},$$

(12.3) $$K_{k}=P_{k,k-1}H_{k}^{T}{\left(H_{k}P_{k,k-1}H_{k}^{T}+R_{k}\right)}^{-1},$$

where ${\hat{X}}_{k,k-1}$ is the priori state estimate, $K_{k}$ is the optimal Kalman gain, $P_{k,k-1}=E\left[{\hat{X}}_{k,k-1}{\hat{X}}_{k,k-1}^{T}\right]$ is the priori error covariance matrix, $P_{k}=E\left[{\hat{X}}_{k}{\hat{X}}_{k}^{T}\right]$ is the posteriori error covariance matrix. The items $P_{k,k-1}$ and $P_{k}$ are computed as,

(12.4) $$P_{k,k-1}=F_{k,k-1}P_{k-1}F_{k,k-1}^{T}+G_{k-1}Q_{k}G_{k-1}^{T},$$

(12.5) $$P_{k}=\left(I-K_{k}H_{k}\right)P_{k,k-1},$$

The item ${\hat{X}}_{k}$ is the optimal estimate of the errors of position, velocity and the IMU errors. It also used to compensate the position, velocity and IMU errors. In the normal case, the item ${\hat{X}}_{k}$ is close to zero, meaning that the all errors of INS are compensated. According to the errorless ${\hat{X}}_{k}$, the item $Z_{k}$ equal to the GNSS noise, and $E\left(Z_{k}\right)=0$. However, when the standard Kalman Filer is in steady state, the matrix $P_{k,k-1}$, $P_{k}$, and $K_{k}$ tend to be constant matrices. They slightly vary with the change of measurement noise or process noise. As a result, the Kalman Filter is degraded and equivalent to a constant weighted average method.

Under a spoofing attack, the GNSS position and velocity errors include additional spoofing-induced relative position and relative velocity. The measurement $Z_{k}$ becomes,

(13) $$Z_{k}^{J}=\left[\begin{array}{c} \delta P_{k}+N_{g,k}+∆p_{s,k}\\ \delta V_{k}+M_{g,k}+∆v_{s,k} \end{array}\right]=Z_{k}+J_{k},$$

where the spoofing vector $J_{k}$ is ${\left[\begin{array}{cc} ∆p_{s,k} & ∆v_{s,k} \end{array}\right]}^{T}$. The offsets of position $∆p_{s,k}$ and velocity $∆v_{s,k}$ generated by spoofing attacks cannot be eliminated by using Equation (11). The spoofed measurements $Z_{k}^{J}$ includes the deviation $J_{k}$. Clearly, spoofing attracts can lead to measurement increase. Besides spoofing attacks, the increase may result from the vehicle motion and IMU error.

Substituting Equation (13) into (12.1), the state compensation ${\hat{X}}_{k}^{J}$ at moment *k* when spoofing attack appears becomes,

(14) $${\hat{X}}_{k}^{J}={\hat{X}}_{k,k-1}+K_{k}\left(Z_{k}^{J}-H_{k}{\hat{X}}_{k,k-1}\right)={\hat{X}}_{k}+K_{k}J_{k}={\hat{X}}_{k}+\delta {\hat{X}}_{k}^{J},$$

In Equation (14), the priori state estimate ${\hat{X}}_{k,k-1}$ is related to the previous state compensation ${\hat{X}}_{k-1}$, which is independent of the measurement $Z_{k}^{J}$ at the *k* − 1 moment, and ${\hat{X}}_{k}$ is the part of state compensation without spoofing attack effects. Therefore, the spoofing effects on state compensation is $\delta {\hat{X}}_{k}^{J}=K_{k}J_{k}$. Since $J_{k}\neq 0$ and $E\left(\delta {\hat{X}}_{k}^{J}\delta {\hat{X}}_{k}^{J,T}\right)>0$, it can be obtained that $E\left({\hat{X}}_{k}^{J}{\hat{X}}_{k}^{J,T}\right)>E\left({\hat{X}}_{k}{\hat{X}}_{k}^{T}\right)=P_{k}$. The value of $P_{k}$ in the spoofing environment varies slightly, disagreement with the state error variations. As pointed in Reference [18], it is difficult to detect spoofing attacks using single $P_{k}$. Meanwhile, the posteriori state estimate ${\hat{X}}_{k}$ varies with $J_{k}$. It means that the spoofing attack causes abnormal corrections on all state compensations, including the navigation compensations (δP and δV) and IMU error compensations ($\varepsilon_{b}$, $\varepsilon_{r}$ and ∇). Between the two types of compensation, the navigation information follows the spoofed GNSS [18]; because, without spoofing detection, integration algorithms tend to believe the position and velocity difference between GNSS and INS relating to the carrier motion. Thus, the position and velocity from the integration system are quickly corrected as the similar to the spoofing values.

The IMU errors refer to its inherent physical characteristics, and therefore the compensation of the IMU errors $\varepsilon_{b}$, $\varepsilon_{r}$ and ∇ theoretically vary within reasonable ranges. During spoofing, GNSS measured position and velocity are inconsistent with INS estimated ones. The inconsistency leads to abnormal compensation of the IMU errors through the Kalman gain.

Under MEAC attacks $J_{k}={\left[∆p_{s,k}^{T},0_{3\times 1}\right]}^{T}$, the item $\delta {\hat{X}}_{k}^{J}$ is $K_{k,p}^{PV}∆p_{s,k}$ for the integration system with position/velocity fusion, and $\delta {\hat{X}}_{k}^{J}=K_{k,p}^{P}∆p_{s,k}$ for the system with position fusion. The IMU error compensations are the 7–15th elements of $\delta {\hat{X}}_{k}^{J}$ and their values are proportional to $∆p_{s,k}$. Both integration systems, with position and position/velocity fusions, are sensitive to the spoofing position deviations under MEAC attacks. Under LOA attack, the spoofing position gradually deviates from the authentic position with small initial position offset $∆p_{s,0}\approx 0$ ($J_{k}$=${\left[\begin{array}{cc} 0_{3\times 1} & ∆v_{s,k} \end{array}\right]}^{T}$). The state compensation is $\delta {\hat{X}}_{k}^{J}=K_{k,p}^{P}0=0$ in the system with position fusion and the norm $\|K_{k,p}^{P}∆p_{s,k}\|=0$. In the system with position/velocity fusion, the item $\delta {\hat{X}}_{k}^{J}=K_{k,v}^{PV}∆v_{s,k}\neq 0$, is proportional to spoofing velocity deviations $∆v_{s,k}$. Therefore, the IMU error compensations in the system with position/velocity fusion have more significant increment at the moment spoofing velocity changes.

Generally, the analysis shows three effects of spoofing attacks on the GNSS/INS loosely coupled integration systems.

(1) Spoofing attack $J_{k}={\left[\begin{array}{cc} ∆p_{s,k} & ∆v_{s,k} \end{array}\right]}^{T}$ leads to an additional compensation $\delta {\hat{X}}_{k}^{J}=K_{k}J_{k}$ in the integration systems. The $\delta {\hat{X}}_{k}^{J}$ modifies the INS estimated position and velocity into spoofed ones.
(2) The $\delta {\hat{X}}_{k}^{J}$ mis-corrects the IMU errors different from its own physical features, which is possibly used to detect spoofing attacks.
(3) The integration systems with position fusion and position/velocity fusion are susceptible to MEAC attacks. To LOA attack, the system with position/velocity fusion is more susceptible.

## 4. Experimental Results and Discussions

In this section, MEAC attack and LOA attack are implemented. The performances of the Global Positioning System/Inertial Navigation System (GPS/INS) loosely coupled integration systems with position fusion and position/velocity fusion are tested under these two spoofing attacks.

### 4.1. Experimental

**Figure 2:**
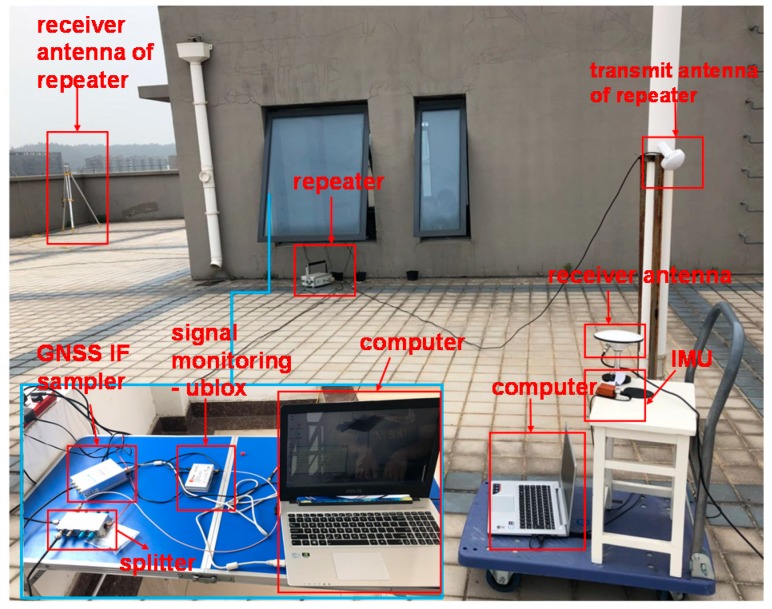
Experimental setup of spoofing attacks on the roof of CEA Building 2.

Figure 2 shows the experimental setup of the spoofing interference on the roof of the College of Automation Engineering (CAE) Building 2 in the Nanjing University of Aeronautics and Astronautics (NUAA) campus.

A GPS repeater is used to generate spoofing signal. Its receiving antenna is about 20 m distance from the target receiver in the case of MEAC attack, and gradually far away from the target receiver in the case of LOA attack. In the test, the spoofing signals cover limited ranges, about 2–5 m. The transmitting antenna should be close to the target receiver to ensure a strong power of spoofing signals. The target receiver is a software-defined GPS receiver with the OLinkStar NS210M IF sampler (OLinkStar Co., Beijing, China) collecting GPS L1 signals. Meanwhile, inertial data are acquired using the low-accurate Xsens MTi-G-710 inertial sensors (Xsens Co., Enschede, The Netherlands), where nominal specifications are shown in Table 1.

It should be noted that spoofing attacks in the experiments are not strict MEAC attack or LOA attack, due to the unknown hardware delay of the repeater. The two cases simulate the position and velocity variations of MEAC attack and LOA attack. LOA attack tries to drag the GPS measured position gradually far away from its authentic position. MEAC attack tends to lead a sudden position variation. In MEAC attack case, the distance between antennas of the receiver and repeater is fixed. The collected data length is about 150 s and spoofing attack starts at 83 s. In LOA attack case, the antenna of the repeater is slowly (about 1.2 m/s measured by GPS) away from the receiving antenna of the receiver.

Two common loosely coupled integration systems are tested. One integration system fuses position and velocity information in the Kalman Filter; the other uses position information only. The employed standard Kalman Filter is updated twice per second. The measurement noise covariance matrix is set as $R_{k}=\mathrm{diag}{\left(\left[\begin{array}{cc} \begin{array}{ccc} 3\;m & 3\;m & 6\;m \end{array} & \begin{array}{ccc} 0.2\;m/s & 0.2\;m/s & 0.2\;m/s \end{array} \end{array}\right]\right)}^{2}$, based on the accuracy of GPS position errors and velocity errors in ENU coordinate. The process noise, is set as $Q_{k}=E\left(\left[\begin{array}{ccc} w_{wg} & w_{rg} & w_{wa} \end{array}\right]{\left[\begin{array}{ccc} w_{wg} & w_{rg} & w_{wa} \end{array}\right]}^{T}\right)$, where $w_{wg}=\left[\begin{array}{ccc} 0.28 & 0.28 & 0.28 \end{array}\right]$ deg/s, $w_{rg}=\left[\begin{array}{ccc} 10 & 10 & 10 \end{array}\right]\;$ deg/h, and $w_{wa}=\left[\begin{array}{ccc} 98 & 98 & 98 \end{array}\right]\;$μg.

### 4.2. Navigation Performanceof the GPS/INS under SpoofingAttacks

**Figure 3:**
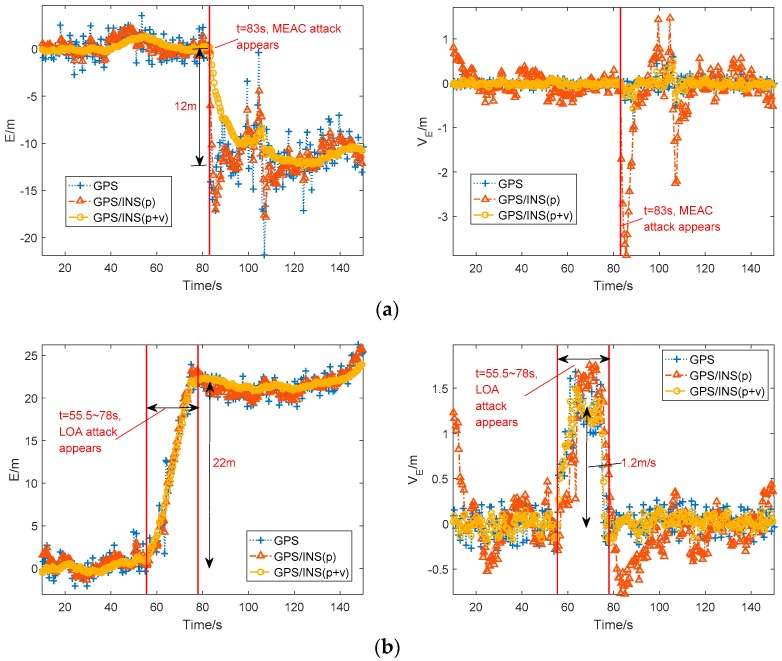
Eastern position error and eastern velocity estimated by Global Positioning System (GPS) and Global Positioning System /Inertial Navigation System (GPS/INS) under Meaconing attacks (MEAC) attack (**a**) and lift-of-aligned (LOA) attack (**b**). MEAC attack begins at 83 s and LOA attack is during 55 s to 78 s.

The average position in East-North-Up coordinate from the GPS receiver under normal situation is considered as the reference value. Figure 3 shows the eastern position errors and eastern velocities from the GPS receiver and the two GPS/INS loosely coupled integration systems.

In Figure 3a, during MEAC attack, the eastern position errors of GPS suddenly jump to −12 m from 0 m and the eastern velocity mainly keeps at zero except a slight increment during 83 s to 90 s. The slight increment on velocity probably results from spoofing attack. The eastern position errors from the two GPS/INS loosely coupled integration systems show the similar performance to that of GPS. Although the velocity fusion smooths the increase of position error, the GPS/INS system has been spoofed under MEAC attack. For the velocity estimation, the integration system with position/velocity fusion performs similar to the GPS receiver, estimated eastern velocity varying slightly and close to zero. However, the integration system with position fusion displays a high sensitivity to MEAC attack, a −3.8 m/s eastern velocity jerk following the occurrence of MEAC attack.

As shown in Figure 3b, under LOA attack, the eastern position errors of GPS deviate gradually from the authentic location during 55.5 s to 78 s, and maintain at about 22 m after 78 s. Like the GPS receiver, the both integration systems are spoofed by LOA attack. Their velocity estimations show the same variation during the spoofing period. A short velocity increase by 0.5–1.8 m/s occurs during the dragging period from 55.5 s to 78 s. Then, the velocity turns back zero after 78 s.

To investigate the overall influence of spoofing attacks on the position and velocity, the norms of position compensation ‖δp‖ and velocity compensation ‖δv‖ are defined as:

(15) $$\begin{array}{c} \|\delta p\|=\sqrt{{\left(\delta p_{e}\right)}^{2}+{\left(\delta p_{n}\right)}^{2}+{\left(\delta p_{u}\right)}^{2}}=\sqrt{\sum_{i=1}^{3}{\left({\hat{X}}_{k}^{J}\left(i\right)\right)}^{2}},\\ \|\delta v\|=\sqrt{{\left(\delta v_{e}\right)}^{2}+{\left(\delta v_{n}\right)}^{2}+{\left(\delta v_{u}\right)}^{2}}=\sqrt{\sum_{i=4}^{6}{\left({\hat{X}}_{k}^{J}\left(i\right)\right)}^{2}}, \end{array}$$

**Figure 4:**
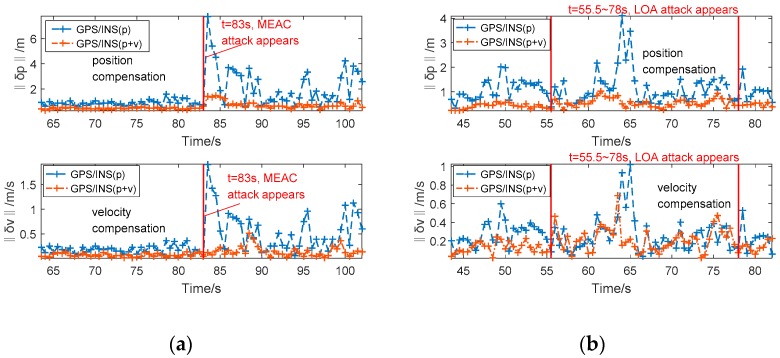
The position and velocity compensation norms estimated by the GPS/INS with position fusion and position/velocity fusion under MEAC attack (**a**) and LOA attack (**b**).

Figure 4 shows the norms of position and velocity compensation under spoofing attacks. Under MEAC attack, the position and velocity compensation norms from the GPS/INS with position fusion increases greatly, while the norms from the GPS/INS with position/velocity fusion change a little. Under LOA attack, both the norms estimated by the GPS/INS with position fusion and position/velocity fusion here have some increments, not as significant as variations under MEAC attack. In addition, the norm increments of position and velocity from the system with position fusion quickly and sharply follow the occurrence of MEAC attack. Under LOA attack, the increment occurs at 64 s, about lagging 9 s to the occurrence of LOA attack. It implies a possible spoofing detection for the integration system with position fusion through using unreasonable increment on norms of position and velocity compensation. The unreasonable norm increment occurs following MEAC attack and a period lagging to LOA attack. It should be pointed out that under LOA attack the position and velocity compensation norms increments is difficult to detect.

Shortly, the GPS/INS loosely coupled integration systems based on standard Kalman Filter are deceived by both MEAC and LOA attacks.

### 4.3. The Variations of IMU Error Compensation under MEAC and LOA Attacks

**Figure 5:**
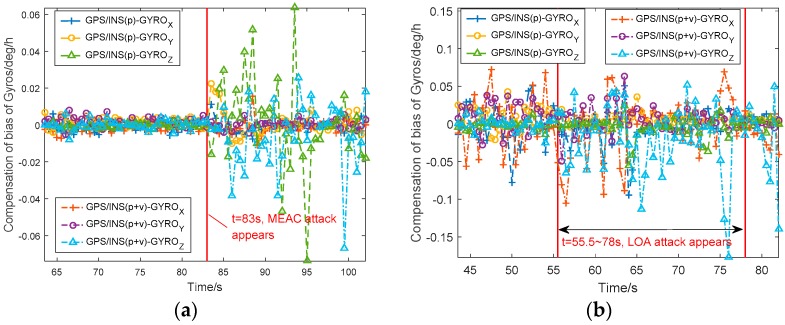
The compensation of gyroscope bias in GPS/INS with position and position/velocity fusions under MEAC attack (**a**) and LOA attack (**b**).

Figure 5 shows the estimated gyroscope bias errors from the GPS/INS with position and position/velocity fusions under MEAC and LOA attacks. Before spoofing, the gyroscope bias errors vary within 0.01 deg/h in Figure 5a and 0.03 deg/h in Figure 5b. Under MEAC attack, the gyroscope bias errors in Z-axis range between −0.07 deg/h and 0.06 deg/h. The two integration systems show the similar performance. The gyroscope bias errors significantly increase during the whole MEAC spoofing. Under LOA attack, the gyroscope bias errors estimated by the position/velocity-fusion-based integration system become large, ranging between −0.08 deg/h and 0.14 deg/h. With the position fusion, the GPS/INS integration system is little disturbed by LOA attack.

In addition, the effects of spoofing attacks on different axial gyroscopes are different. To investigate the overall influence of the spoofing attack on the IMU, the norms of the XYZ-axis gyroscope bias compensation $\left\|\varepsilon_{b}\right\|$, the gyroscope first-order Markov compensation $\left\|\varepsilon_{r}\right\|$ and the XYZ-axis accelerometers bias compensation ‖∇‖ are defined as,

(16) $$\begin{array}{c} \|\varepsilon_{b}\|=\sqrt{{\left(\varepsilon_{bx}\right)}^{2}+{\left(\varepsilon_{by}\right)}^{2}+{\left(\varepsilon_{bz}\right)}^{2}}=\sqrt{\sum_{i=7}^{9}{\left(\delta {\hat{X}}_{k}^{J}\left(i\right)\right)}^{2}},\\ \|\varepsilon_{r}\|=\sqrt{{\left(\varepsilon_{rx}\right)}^{2}+{\left(\varepsilon_{ry}\right)}^{2}+{\left(\varepsilon_{rz}\right)}^{2}}=\sqrt{\sum_{i=10}^{12}{\left(\delta {\hat{X}}_{k}^{J}\left(i\right)\right)}^{2}},\\ \|\nabla \|=\sqrt{{\left(\nabla_{x}\right)}^{2}+{\left(\nabla_{y}\right)}^{2}+{\left(\nabla_{z}\right)}^{2}}=\sqrt{\sum_{i=13}^{15}{\left(\delta {\hat{X}}_{k}^{J}\left(i\right)\right)}^{2}}, \end{array}$$

**Figure 6:**
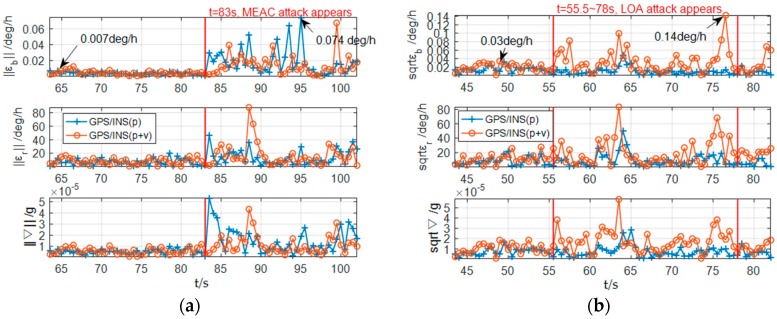
The IMU error compensation norms estimated by GPS/INS with position and position/velocity fusions under MEAC attack (**a**) and LOA attack (**b**).

Figure 6 shows the norms of the IMU error compensation under spoofing attacks. Under MEAC attack, as shown in Figure 6a, the norms of the gyroscope bias compensations become much larger than that under spoofing-free period. The norm of the gyroscope bias compensation reaches 0.074 deg/h at 95 s during spoofing period. Under spoofing-free condition, the maximum norm value is 0.007 deg/h at 64.5 s. Similar variations are also found in $\left\|\varepsilon_{r}\right\|$ and ‖∇‖. Although the occurrence of the incorrectness in the GPS/INS loosely coupled integration with position/velocity fusion is 1 s slower than that in the system with position fusion, two systems show the similar sensitivity to MEAC attack. Thus, the norms of the IMU error compensation have the capability to detect MEAC attack.

Under LOA attack, as shown in Figure 6b, the increments of $\left\|\varepsilon_{b}\right\|$, $\left\|\varepsilon_{r}\right\|$ and ‖∇‖ from the integration system with the position fusion become insignificant. Differently, from the system with the position/velocity fusion, the norms also increase during LOA attack. Although the increments are not as obvious as that under MEAC attack, the maximum value of $\left\|\varepsilon_{b}\right\|$ reaches 0.14 deg/h, 4.6 times larger than the value under spoofing-free periods.

**Figure 7:**
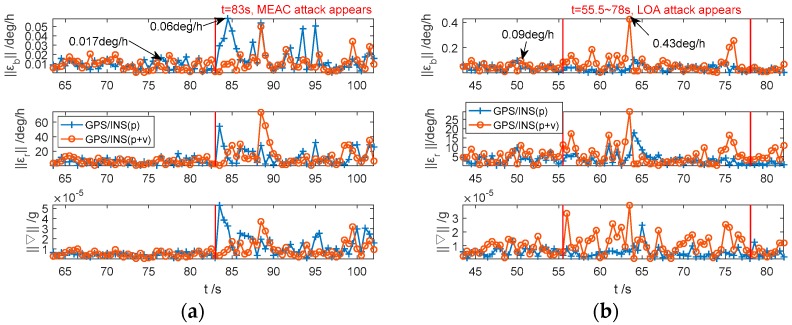
The IMU error compensation norms estimated by the GPS/INS with position and position/velocity fusions under MEAC attack (**a**) and LOA attack (**b**) in the dynamic case.

To test the performance of $\left\|\varepsilon_{b}\right\|$, $\left\|\varepsilon_{r}\right\|$ and ‖∇‖ under dynamic situation, the IMU is placed on a turntable to simulate attitude movement, i.e., heading, pitch and roll angle changes simultaneously. The collected inertial data is integrated with the spoofed GPS data to study whether spoofing attacks would have significant impacts on the dynamic IMU error compensation. The results are shown in Figure 7.

In Figure 7a, the fluctuations of IMU error compensation norms, under spoofing-free period, are slightly larger than the static IMU. Under MEAC attack, the norms significantly increase regardless of whether the fusion information is position or position/velocity. Similar to the static case, the variation of $\left\|\varepsilon_{b}\right\|$ from the integration system with position fusion is not obvious under LOA attack, as shown in Figure 7b. With the position/velocity fusion, the maximum value of $\left\|\varepsilon_{b}\right\|$ reaches 0.43 deg/h during spoofing period, which is five times of the maximum norm under normal condition. The variations of $\left\|\varepsilon_{r}\right\|$ and ‖∇‖ are similar to $\left\|\varepsilon_{b}\right\|$. It should be noted that the moment with obvious spoofing-induced increments on $\left\|\varepsilon_{b}\right\|$, $\left\|\varepsilon_{r}\right\|$ and ‖∇‖ are later than the moment spoofing appearing. It may be due to the smoothing effects of Kalman Filter on velocity correction.

Shortly, for the loosely coupled integration system with the position/velocity fusion, the IMU error compensations are sensitive to both MEAC and LOA attacks. The compensations increase significantly during spoofing. For the system with position fusion, the IMU error compensation are sensitive to MEAC attacks. Different to the position and velocity compensations which also affected by the receiver dynamic situation, the IMU error compensations refer to IMU physical features and are possible to detect spoofing attacks in the GPS/INS loosely coupled integration systems.

**Figure 8:**
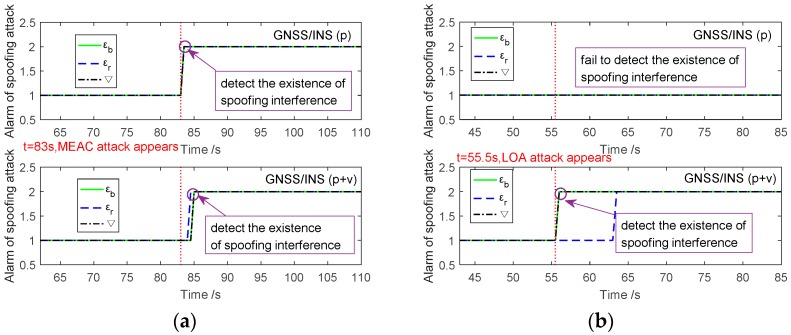
The detection of spoofing attack based on the IMU error compensation norms under MEAC attack (**a**) and LOA attack (**b**).

To test the feasibility of spoofing attack detection based on IMU error compensations, a basic detection is employed. When the instantaneous $\left\|\varepsilon_{b}\right\|$ is larger than its historical statistics, the state of $\left\|\varepsilon_{b}\right\|$ will stay at pre-alarm state (marked as 2 in Figure 8), which means there would be a possibility of spoofing attack. The states of the item $\left\|\varepsilon_{r}\right\|$ and ‖∇‖ are detected using the similar method. Then the alarm of spoofing attack is given through combining detection results of $\left\|\varepsilon_{b}\right\|$, $\left\|\varepsilon_{r}\right\|$ and ‖∇‖. Since both $\left\|\varepsilon_{b}\right\|$ and $\left\|\varepsilon_{r}\right\|$ are the features of gyroscopes and ‖∇‖ is related to accelerometers, the spoofing attack is alarmed when the state of ‖∇‖ is pre-alarm, and any state of $\left\|\varepsilon_{b}\right\|$ or $\left\|\varepsilon_{r}\right\|$ is pre-alarm.

**Figure 9:**
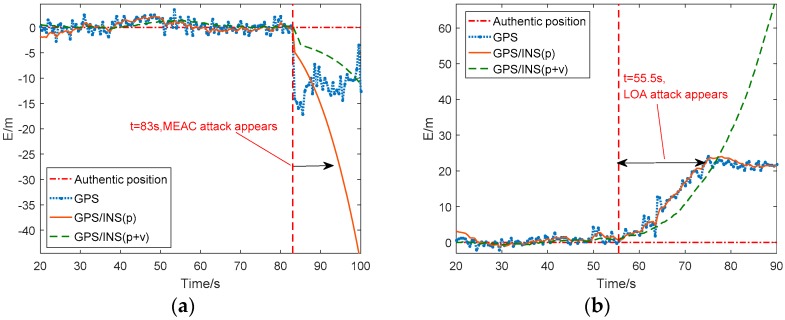
The performance of GPS/INS with position and position/velocity fusions after detection of MEAC attack (**a**) and LOA attack (**b**).

Figure 8a illustrates the detection results of MEAC attack in GPS/INS with position and position/velocity fusions. Under MEAC attack, $\left\|\varepsilon_{b}\right\|$, $\left\|\varepsilon_{r}\right\|$ and ‖∇‖ of the GNSS/INS with position fusion suddenly rise at 83 s, the moment of MEAC attack beginning. All $\left\|\varepsilon_{b}\right\|$, $\left\|\varepsilon_{r}\right\|$ and ‖∇‖ are into the pre-alarm state and spoofing is detected at 83 s. For the GPS/INS with position/velocity fusion, the visible rise of $\left\|\varepsilon_{b}\right\|$, $\left\|\varepsilon_{r}\right\|$ and ‖∇‖ follows the occurrence of MEAC attack after about two seconds. Each of them alarms the spoofing after a short delay. Therefore, the voted alarm of MEAC attack is about two second later than the moment of spoofing occurrence. By contrast, under LOA attack, as shown in Figure 8b, any item of $\left\|\varepsilon_{b}\right\|$, $\left\|\varepsilon_{r}\right\|$ and ‖∇‖ in system with position fusion fails in alarming spoofing; because, the IMU error compensations increase slightly. Fortunately, these items, especially the ‖∇‖ and $\left\|\varepsilon_{b}\right\|$, of the system with position/velocity fusion, increase dramatically once LOA attack occurs. Although the item $\left\|\varepsilon_{r}\right\|$ switches into pre-alarm state with 8 s delay, the voted spoofing alarm is at 56 s, close to the occurrence moment of LOA attack. In short, INS error compensations, in both integration systems with position and position/velocity fusions, can be used to detect MEAC attack. LOA attack is likely to be detected effectively by using the INS error compensations from the position/velocity fusion-based integration system. Once the spoofing attack being detected, it is suggested to reject GPS information input to Kalman filter, which is shown in Figure 9.

Figure 9 shows the eastern position error estimated by GPS/INS with two types of fusions before and during spoofing attacks. Under MEAC attack, as shown in Figure 9a, both GPS/INS systems with position fusion and position/velocity fusion succeed in detecting the occurrence of MEAC attack with a short delay, about one or two seconds. The integration system degrades into independent INS. The estimated position is no longer deceived by spoofing attacks. It is no doubt that the position error of stand-alone INS increases with time. Therefore, in Figure 9a, the position error of GPS/INS with position fusion increases to 12 m at 88 s. After 5 s since spoofing detection, the position error of the system with position fusion is larger than the spoofing induced position error. With position/velocity fusion, the GPS/INS shows better performances than the one with position fusion. It keeps its position error within 12 m for about 16 s. The improvement is probably due to more accurate error compensation than that estimated by the system with position fusion. Under LOA attack, as shown in Figure 9b, the GPS/INS with position fusion fails in spoofing detection since its $\left\|\varepsilon_{b}\right\|$, $\left\|\varepsilon_{r}\right\|$ and ‖∇‖ increments are too slight to detect spoofing attack. The system with position/velocity fusion still successfully detects the spoofing attack and turns into stand-alone INS at 56 s. The position error under LOA attack quickly increases to 22 m at 76 s. During the 20 s duration, the INS position error keeps less than spoofing induced position error. The slight superiority results from the growth rate of INS position error lower than the spoofing velocity $\Delta v_{s}$. It is a combination result of IMU performance and error compensation, as well as the values of spoofing velocity $\Delta v_{s}$ and position deviation $\Delta p_{s}$, which are out of the discussion of the study.

Shortly, IMU error compensation-based spoofing detection is effective. For GPS/INS with position/velocity fusion, it is possible to detect both MEAC attack and LOA attack. Once the integration system turns into the stand-alone INS after spoofing detection, the system is able to isolate spoofing effects. In this case, the accumulative position error is inevitable. High-precise IMUs and advanced IMU error compensation algorithms are helpful in slowing the INS position error growth.

## 5. Conclusions

In this paper, the performance of the GNSS/INS loosely coupled integration systems with position and position/velocity fusions under MEAC attacks and LOA attacks are analyzed. The GNSS/INS loosely coupled integration systems with either position fusion or position/velocity fusion is easily disturbed by spoofing attacks, similar to GNSS.

However, we can exploit the fact that the position and velocity from GNSS are inconsistent with the physical states measured by the INS. The inconsistency causes abnormal corrections to the IMU error compensations. Specially, the incorrect IMU error compensations from the integration system with the position fusion are significant under MEAC attack, which generates a jump of positioning results. Under LOA attack with slow position variation, as well as MEAC attacks, the compensations from the system with the position/velocity fusion are more sensitive. Thus, it is possible, using IMU error compensations, which are related to IMU’s physical features, to detect spoofing attacks. A simple detection method is implemented and tested. The detection results show that with position/velocity fusion, the GNSS/INS loosely coupled integration system is able to detect both MEAC and LOA spoofing attacks through using the IMU error compensations.

The paper focuses on the possibility of spoofing detection based on IMU error compensation for the loosely coupled integration system. Further studies will interest in the effects of IMUs with different accuracy levels and the spoofing detection and mitigation methods.

## Figures and Tables

**Table 1:** IMU nominal specifications.

	Gyro	Accelerometer
In-run bias stability	10 deg/h	40 μg
Noise density	0.01 deg/s/Hz^1/2^	80 μg/Hz^1/2^
